# Divergent expression patterns of pituitary gonadotropin subunit and GnRH receptor genes to continuous GnRH *in vitro* and *in vivo*

**DOI:** 10.1038/s41598-019-56480-1

**Published:** 2019-12-27

**Authors:** Marija M. Janjic, Rafael M. Prévide, Patrick A. Fletcher, Arthur Sherman, Kosara Smiljanic, Daniel Abebe, Ivana Bjelobaba, Stanko S. Stojilkovic

**Affiliations:** 1 Eunice Kennedy Shriver National Institute of Child Health and Human Development, National Institutes of Health, Bethesda, MD 20892 USA; 2 Institute for Biological Research Sinisa Stankovic - National Institute of Republic of Serbia, University of Belgrade, 11000 Belgrade, Serbia; 3 Laboratory of Biological Modeling, National Institute of Diabetes, Digestive and Kidney Diseases, National Institutes of Health, Bethesda, MD 20892 USA

**Keywords:** Peptide hormones, Pituitary gland

## Abstract

Continuous, as opposed to pulsatile, delivery of hypothalamic gonadotropin-releasing hormone (GnRH) leads to a marked decrease in secretion of pituitary gonadotropins LH and FSH and impairment of reproductive function. Here we studied the expression profile of gonadotropin subunit and GnRH receptor genes in rat pituitary *in vitro* and *in vivo* to clarify their expression profiles in the absence and continuous presence of GnRH. Culturing of pituitary cells in GnRH-free conditions downregulated *Fshb*, *Cga*, and *Gnrhr* expression, whereas continuous treatment with GnRH agonists upregulated *Cga* expression progressively and *Gnrhr* and *Fshb* expression transiently, accompanied by a prolonged blockade of *Fshb* but not *Gnrhr* expression. In contrast, *Lhb* expression was relatively insensitive to loss of endogenous GnRH and continuous treatment with GnRH, probably reflecting the status of Egr1 and Nr5a1 expression. Similar patterns of responses were observed *in vivo* after administration of a GnRH agonist. However, continuous treatment with GnRH stimulated LH secretion *in vitro* and *in vivo*, leading to decrease in LH cell content despite high basal *Lhb* expression. These data suggest that blockade of *Fshb* expression and depletion of the LH secretory pool are two major factors accounting for weakening of the gonadotroph secretory function during continuous GnRH treatment.

## Introduction

Mammalian reproduction depends on proper synthesis and release of two gonadotropins, luteinizing hormone (LH) and follicle-stimulating hormone (FSH), by specialized endocrine cells of the anterior pituitary named gonadotrophs^1^. These hormones are dimeric glycoproteins composed of a common glycoprotein hormone, α polypeptide (Cga) and unique β subunits (Lhb and Fshb) that confer biological specificity^2^. The secretion of gonadotropins is tightly regulated by hypothalamic and intrapituitary factors. Among them, the most important is gonadotropin-releasing hormone (GnRH), which is released in a pulsatile manner by a small set of neurons within the preoptic area and mediobasal hypothalamus^3^. Upon reaching the anterior pituitary, GnRH binds to its receptor expressed in gonadotrophs and signals through a G_q/11_-dependent cascade of intracellular pathways that culminates in periodic gonadotropin secretion^4^.

The pulsatile pattern of GnRH release is critical for proper gonadotropin synthesis and release. This was initially established in rhesus monkeys with hypothalamic lesions, which abolish endogenous GnRH secretion, where blood levels of LH and FSH were undetectable. Reintroduction of pulsatile GnRH administration rescued gonadotropin secretion, and subsequent continuous GnRH infusion lead to marked decrease in blood LH and FSH levels^5^. Subsequent experiments by the same group also revealed that LH levels were maximally stimulated at a higher GnRH pulse frequency than FSH^6^. The requirement of pulsatile GnRH release for the proper reproductive function was subsequently confirmed in other mammalian species^3^. Several physiological responses and pathological conditions that cause reproductive failure in humans are also associated with dysregulation of pulsatile GnRH release, including functional hypothalamic amenorrhea, hyperprolactinemia, polycystic ovary syndrome, and hypogonadotropic hypogonadism^7–10^. Continuous GnRH receptor agonist treatments are also clinically relevant, for example, for prevention of ovarian hyperstimulation syndrome during assisted reproduction^11,12^, for ovarian protection during chemotherapy^13^, and for treatments of precocious puberty^14^ and polycystic ovary syndrome^15^.

In general, the gonadotroph subunit genes, *Cga*, *Fshb*, and *Lhb*, exhibit basal and regulated expression, and it has been shown that they are stimulated by pulsatile GnRH application^1^. For example, *in vivo* pulsatile application of GnRH in male rats^16^, and *in vitro* pulsatile delivery of GnRH to cultured pituitary cells from male rats^17^ and pituitary fragments from female rats^18^ increased the transcription of gonadotropin subunit genes. In parallel to serum gonadotropin levels, *Fshb* was found to be preferentially transcribed at lower GnRH pulse frequencies, whereas *Lhb* was suggested to be preferentially transcribed at higher pulse frequencies administrated to male rats^16,19^ and to cultured pituitary cells from male rats^17^. Thus, the transcriptional regulation of gonadotropin subunit genes by pulsatile GnRH could be a critical point that determines gonadotropin biosynthesis and secretion, suggesting the hypothesis that continuous GnRH treatment may cause downregulation of gonadotropin subunit gene expression, leading to downregulation of gonadotropin release.

However, the mechanism of downregulation of gonadotropin release by continuous GnRH treatment has not been clarified. Shupnik’s group observed stimulation of *Fshb* and *Lhb* transcription in static cultures of pituitary fragments from female rats during the first 6 h continuous application of GnRH, but they did not detect inhibition of basal expression of these genes^18^. The same group also reported lack of changes in *Cga*, *Lhb*, and *Fshb* transcription compared to controls after continuous *in vivo* application of GnRH for 4 h in male rats^16^. In perifused rat pituitary cells, *Fshb* expression was stimulated by pulsatile delivery of GnRH for 12 h, but inhibited by continuous GnRH application, whereas *Lhb* mRNA levels were unresponsive to either mode of GnRH application^20^. The intrapituitary *Fshb* expression and circulating FSH levels are often highly correlated, and FSH was suggested to be secreted by constitutive exocytosis^21^ (and references within). In contrast, LH is secreted by regulated exocytosis^22^, i.e. GnRH receptor controls *Lhb* expression, LH release, or both.

Here, we evaluated the time course of gonadotropin subunit gene expression in static cultures of rat anterior pituitary during continuous GnRH treatment. We also studied the expression of transcription factor genes *Egr1*, *Nr5a1*, and *Pitx1*, which are critically important for control of expression of *Lhb*^23–25^. In addition, we characterized the acute and long-term effects of cell dispersion and primary culture in the absence of GnRH for these genes and commonly used reference genes before examining the effect of continuous GnRH treatment. Furthermore, we studied the relationship between *Lhb* expression, LH cell content, and LH release *in vitro* during continuous GnRH treatment. Finally, we evaluated effects of *in vivo* injection of a GnRH receptor agonist and antagonist on expression of gonadotropin subunit genes and LH serum and pituitary content. The expression of *Gnrhr* and *Dmp1*, which we characterized recently^26,27^, were used as internal controls in both *in vitro* and *in vivo* experiments. Our results indicate that continuous GnRH application progressively stimulates *Cga* expression, blocks *Fshb* expression after a transient stimulation, and causes a profound effect on LH secretion without major effects on *Lhb* expression.

## Methods

### Animals and experimental procedures

*In vitro* experiments were performed with anterior pituitary cells derived from 8-12-week-old Sprague Dawley rats obtained from Taconic Farms (Germantown, NY). Animals were euthanized via asphyxiation with CO_2_. After decapitation anterior pituitary glands were removed and cells were mechanically dispersed after trypsin and EDTA treatments as previously described^28^. If not otherwise specified, experiments were performed with cells from females. Cells were seeded on poly-D-lysine coated 24-well plates and initially cultured in medium 199 containing Earle’s salts, sodium bicarbonate, penicillin (100 units per ml), streptomycin (100 µg per ml) and 10% heat-inactivated horse serum (HS) (Life Technologies, Grand Island, NY). After overnight incubation, cells were bathed in medium 199 Earle’s salt solution containing 0.1% BSA in the presence of GnRH or D-Ala6-GnRH (D-Ala6) (Sigma, St Louis, MO). At the end of the experiments, incubation medium was collected, centrifuged at 3000 rpm to remove cell debris, and the supernatant was collected and stored at −80 °C for determining LH concentration. Attached cells were scraped for RNA extraction or LH measurement. For *in vivo* experiments, 4-week old animals were injected once intraperitoneally with a GnRH receptor agonist, buserelin acetate (5 µg/0.4 ml/per animal) from Sigma (St. Louis, MO), a GnRH receptor antagonist, cetrorelix acetate (100 µg/0.4 ml/per animal) from Tocris (Minneapolis, MN), or vehicle (0.4 ml/per animal). Euthanasia was performed 0.5, 1, 1.5, 2, 3, 6, or 9 hours after intraperitoneal injections. After decapitation, blood was collected, and serum was separated and stored at −80 °C for LH concentration measurement. Whole anterior pituitaries were individually collected in RNA stabilization solution (Thermo Fisher Scientific, Waltham, MA) for RNA extraction or individually frozen without solution for measurement of LH tissue content (as described below). All experimental procedures were in accordance with the National Institutes of Health Policy Manual 3040-2: Animal Care and Use in the Intramural Program and were approved by the National Institute of Child Health and Human Development, Animal Care and Use Committee (Animal Protocol 16-041).

### LH measurements

Concentration of LH in samples was determined using an LH Rodent Elisa Kit (Endocrine Technologies, Newark, CA), according to the manufacturer instructions. Samples (blood serum, incubation medium, pituitary tissue content and cell content) were diluted appropriately if needed. For LH tissue content measurements, pituitaries were homogenized in 20 mM sodium carbonate. The homogenate was centrifuged (3000 rpm) and supernatant was transferred to a new vial. Protein concentration was then measured by the Pierce^TM^ BCA assay (Thermo Fisher Scientific, Waltham, MA) and samples were equalized to contain the same total protein concentration prior to Elisa LH measurement.

### qRT-PCR analysis

Total RNA was extracted from individual anterior pituitary glands and primary cultures of anterior pituitary cells using an RNeasy Plus Mini Kit (Qiagen, Valencia, CA). RNA was reverse transcribed with a Transcriptor First Strand cDNA Synthesis Kit (Roche Applied Sciences, IN). An analysis of relative gene expression was performed using qRT-PCR and the comparative C_t_ method. For this, the LightCycler TaqMan Master Mix and the Lightcycler 2.0 Real-time PCR system (Roche Applied Science, Indianapolis, IN), or Power SYBR Green Master Mix and Quant Studio 3 system (Thermo Fisher Scientific, Waltham, MA) were used. Applied Biosystems predesigned TaqMan Gene Expression Assays were used: *Cga*: Rn01440184_m1, *Lhb*: Rn00563443_g1, *Fshb*: Rn01484594_m1, *Gnrhr*: Rn00578981_m1, *Dmp1*: Rn01450122_m1, and *Gapdh*: Rn01462662_g1, *Rpl19*: Rn00821265_g1, *Rps18:* Rn01428913_gH, *Egr1:* Rn00561138_m1, *Nr5a1:* Rn00584298_m1, and *Pitx1:* Rn 00586187_m1. The primer sequences (Integrated DNA Technologies, Skokie, IL) used in SYBR Green quantitative real-time PCR are listed in Table S1.

To compare the relative expression levels of the transcripts, the levels were normalized against a reference gene, i.e. an internal reaction control that had sequences different than the target^29^. Several reference genes have been suggested for qRT-PCR analysis in anterior pituitary based on previous work with normal and pathological pituitary tissues and dispersed pituitary cells from different laboratories^30–34^. Figs. S1 and S2 show the expression patterns of these genes obtained from our single-cell RNA sequencing analysis of freshly dispersed male and female rat anterior pituitary cells^35^. Out of 19 genes studied, only eight genes were expressed in over 90% of anterior pituitary cells: *Rpl19, Rpl32, Rps18, Gapdh, Rpl4, Rplp0, B2m*, and *Actb*. Among them, only five genes exhibited comparable expression levels in all subpopulations of anterior pituitary cells: *Rpl19, Rpl32, Rps18, Gapdh*, and *Rpl4*. For further testing, we selected *Rpl19, Rps18*, and *Gapdh* because their C_t_ values, estimated in pituitary tissue, were comparable to the *Lhb* C_t_ values: *Rpl19* = 21.46 ± 0.07; *Rps18* = 22.37 ± 0.06; *Gapdh* = 23.85 ± 0.07; and *Lhb* = 22.15 ± 0.11.

To further study the validity of these candidate genes for qRT-PCR analysis of *in vitro* experiments, we examined the time-courses of their expression patterns after cell dispersion. We observed a decay in C_t_ values for all three candidate genes during the first 17 h of incubation, independent of the serum used for culturing (HS, BSA, or fetal bovine serum), suggesting that expression of these housekeeping genes increased after cell dispersion. To illustrate those changes, we used the C_t_ value at the beginning of experiments (zero time point, C_t_(0)) as the reference value, computing the expression at later time points relative to time zero as: 2^−(Ct(t)–Ct(0))^, measured in relative units (RU) (Fig. S3A). After 17 h of incubation, the expression of candidate reference genes stabilized until the end of the experiments, i.e. until 96 h after cell dispersion (Fig. S3B). We also tested whether GnRH affects the expression of these candidate reference genes using female pituitary cells cultured overnight. The following morning, cells were washed and incubated in BSA-containing medium 199 supplemented with 10 nM GnRH for up to 8 h. Under these conditions the C_t_ values for *Gapdh*, *Rpl19*, and *Rps18* did not change during incubation; Fig. S3C shows combined mean values from these experiments. 10 nM GnRH also had no effect on *Gapdh* expression during shorter and longer treatments (Fig. S3D).

Based on these findings, in further studies we used expression relative to time zero as described above to evaluate gene expression during the first 20 h of incubation after cell dispersion and termed such values as relative units (RU). For experiments done after 20 h of GnRH-free preincubation, such as those performed after overnight incubation, we used *Gapdh* and/or *Rpl19* as reference genes for experiments and calculated expression relative to the reference gene as: 2^−(C_t_(target gene) −C_t_(reference gene)) × 100. To normalize values from several independent experiments, we also presented such data as fold change relative to time zero, with initial values shown as one.

### Immunocytochemistry

For immunocytochemical analysis, 50,000/well freshly dispersed anterior pituitary cells were plated on poly-D-lysine coated 8-well multitest slides (MP Biomedicals, Aurora, OH) and cultured overnight. The following morning, cells were treated with 10 nM GnRH for 2, 4, 6 and 8 h. Cells were then washed twice with PBS and fixed with cold 4% formaldehyde solution (Thermo Scientific, Rockford, IL, USA) for 10 min at room temperature. After washing cells three times in PBS, cultures were bathed in medium with 1:200 rabbit anti-Egr1 15F7 (Cell Signaling Technology, Danvers, MA) or 1:100 mouse anti-Nr5a1 N1665 (ThermoFisher Scientific, Waltham, MA) antibodies overnight at 4 °C, then incubated with appropriate secondary antibody (Alexa Fluor 488 donkey anti-mouse or Alexa Fluor 488 goat anti-rabbit; Invitrogen, Carlsbad, CA; 1:1000) for 30 min at room temperature. For double immunostaining, pituitary cells were incubated with guinea pig anti-Lhb (1:250) (obtained from Dr. A. F. Parlow, National Institute of Diabetes and Digestive and Kidney Diseases, National Hormone and Peptide Program, Torrance, CA) overnight at 4 °C, followed by subsequent incubation with a suitable secondary antibody (Alexa Fluor 568 goat anti-guinea pig antibody; Invitrogen, Carlsbad, CA; 1:1000 dilution) for 30 min at room temperature. All antibodies were diluted in staining PBS solution containing 0.2% saponin and 0.5% BSA. Every step of the immunostaining protocol was followed by washing cells three times with PBS. Cells were mounted with Fluoromount-G, with 4′,6-diamidino-2-phenylindole, a fluorescent DNA stain (Invitrogen, Carlsbad, CA). To test the specificity of the reaction, control cells were treated in the same way with the exclusion of primary antibodies (data not shown). All images were acquired on an inverted confocal laser-scanning microscope (LSM 780; Carl Zeiss GmbH, Jena, Germany), using a 63x oil objective. Micrographs were sized, and their brightness and contrast levels adjusted in Fiji. Cells were counted on 5 tile-scan images (3 × 3).

### Statistics

The numerical values are reported as the mean ± SEM from a representative set of at least three similar experiments, or normalized values from three to nine independent experiments per time point. KaleidaGraph (Synergy Software, Reading, Pennsylvania) was used for all calculation and graph presentation. Significant differences between means were determined by a single factor ANOVA or an ANOVA followed by the post hoc Student-Newman-Keuls test.

## Results

### Basal gonadotropin subunit and GnRH receptor gene expression patterns *in vitro*

**Figure 1:**
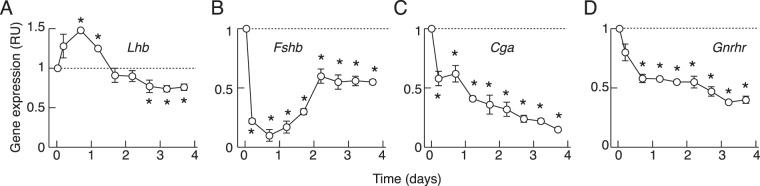
The expression patterns of gonadotropin subunit (**A**–**C**) and GnRH receptor (**D**) genes in rat anterior pituitary cells cultured in the absence of GnRH. Zero time points indicate the beginning of culturing after cell dispersion. Expression was measured relative to time zero in relative units (RU), as described in *Methods: qRT-PCR analysis*. In all panels, asterisks indicate significant difference vs. zero time point controls.

Freshly dispersed rat anterior pituitary cells were plated and cultured in medium without GnRH for four days. Because of transient changes in C_t_ values for *Gapdh* and other candidate reference genes during the first 20 h after cell dispersion, we measured expression relative to expression at time zero (RU), as described in *Methods: qRT-PCR analysis*. Figure 1A shows bidirectional changes in *Lhb* expression after cell dispersion; an initial minor stimulatory effect followed by a minor sustained inhibition. In contrast, there was an initial deep inhibition in *Fshb* expression followed by a partial recovery of expression (B), and a progressive inhibition of *Cga* (C) and *Gnrhr* expression (D). These results show that expression of *Lhb* was relatively insensitive to loss of endogenous GnRH, in contrast to *Fshb*, *Cga*, and *Gnrhr* expression. A partial recovery of *Fshb* expression during prolonged culturing without changes of bath medium could indicate an autocrine/paracrine stimulatory effect.

### Gene-specific expression pattern during continuous GnRH receptor agonists application

**Figure 2:**
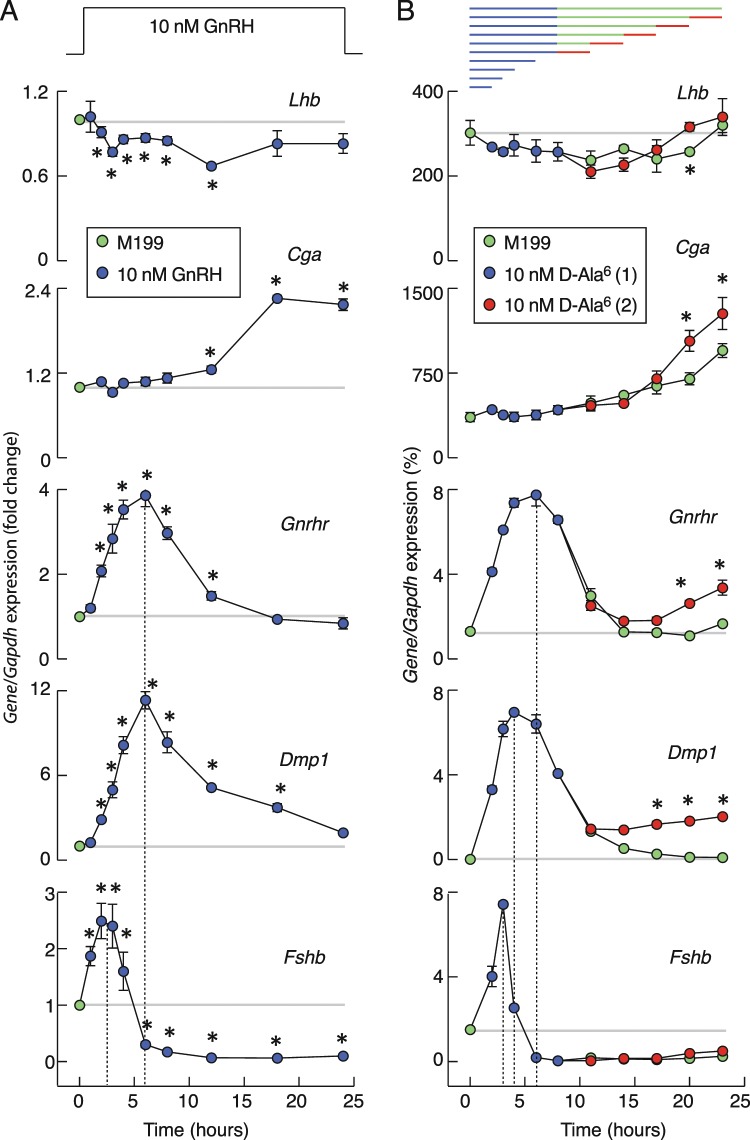
Time-course of GnRH receptor agonists on gonadotropin subunit genes, *Gnrhr*, and *Dmp1* expression in cultured pituitary cells. (**A**) Effects of continuous GnRH application on gene expression. Note the transient nature of *Fshb*, *Gnrhr*, and *Dmp1* expression, a slow and continuous upregulation in *Cga* expression, and a transient and minor inhibition of *Lhb* expression during GnRH treatment. Data shown are mean ± SEM values, normalized to the zero time point controls, from three to nine independent experiments per time point, each performed in quadruplicate. Asterisks indicate significant differences vs. zero time point. (**B**) A gene-specific patterns of expression in response to application of D-Ala6. Top panel indicates duration of first (blue) and second (red) 10 nM D-Ala6 application and washout periods (green). The data shown are mean ± SEM values from a representative experiment; when not visible, SEM values were within circles. Asterisks indicate a significant difference between pairs. In both panels, horizontal gray lines indicate basal gene expression and vertical dotted lines indicate times needed to reach the peak in expression. If not otherwise specified, in this and following figures experiments were performed with cells from postpubertal female rats. Immediately after dispersion, cells (1.5 million per well) were seeded in 24-well plates and cultured overnight in horse serum-containing GnRH-free medium. After 20 h incubation, medium was replaced with fresh 0.1% BSA-containing medium with GnRH or D-Ala6.

In further experiments, anterior pituitary cells were cultured for 20 h after dispersion in the absence of GnRH, washed, and treated with 10 nM GnRH for 1, 2, 3, 4, 6, 8, 12, 18, and 24 h (Fig. 2A). Our control genes for this experiment, *Gnrhr* and *Dmp1*, responded as previously reported^26,27^; their expression was transiently stimulated, reaching a peak within 6 h of stimulation and followed by a gradual decay toward the basal level of gene expression (Fig. 2A). GnRH also stimulated *Fshb* expression transiently but peaking within 2–3 h of stimulation and followed by a rapid decay in gene expression below basal expression. After 6 h of incubation in the presence of GnRH, *Fshb* expression was abolished for the rest of the experiment. GnRH also stimulated *Cga* expression, but with a distinct temporal profile of expression; no changes in expression were observed during the first 8 h of culturing, followed by a slow increase that reached a peak in response after 18 h of stimulation. Finally, we did not observe consistent stimulatory effects of GnRH on *Lhb* expression during continuous application; instead observing only a small and transient inhibition of basal expression during the first 12 h of incubation. Similar patterns of responses were also observed in cultured pituitary cells from postpubertal male rats (data not shown). These data indicate the gonadotropin subunit gene-specific patterns of responses to continuous *in vitro* GnRH application.

The progressive decay in *Fshb, Gnrhr*, and *Dmp1* expression following the peak in response could reflect the rate of downregulation of stimulus-expression coupling in the presence of GnRH and/or the rate of GnRH degradation. To rule out the latter, in further experiments we substituted native GnRH with its degradation-resistant analog D-Ala6, which was applied once or twice during a 23 h period in static cultures of female pituitary cells, as shown on the top panel of Fig. 2B. If applied twice, the culture medium was changed between applications to provide a washout period of variable duration. The profiles of gene expression during the first 12 h of application of D-Ala6 were highly comparable to those seen in GnRH application, with bidirectional effects on *Fshb* expression, stimulatory effect on *Cga* expression, and a small downregulation of *Lhb*. The washout of D-Ala6 did not affect *Fshb* expression during 15 h of incubation, as indicated by the lack of return of expression to the pre-application basal level. Also, the subsequent application of agonist at different time points was unable to elevate expression of this gene. *Lhb* expression recovered with and without secondary application of D-Ala6. The ongoing rise in *Cga* expression observed after removal of D-Ala6 was amplified 34 ± 14% by secondary agonist application. Finally, there was significant recovery of *Gnrhr* expression during the secondary D-Ala6 application, with the amplitude of responses increasing with extension of washout duration. For example, at the end of the experiments, the response of *Gnrhr* was 32 ± 4.5% of that observed during the first D-Ala6 application. These experiments established that continuous GnRH *in vitro* downregulates *Fshb* expression, but not that of *Cga*, *Lhb*, and *Gnrhr*, below basal levels.

**Figure 3:**
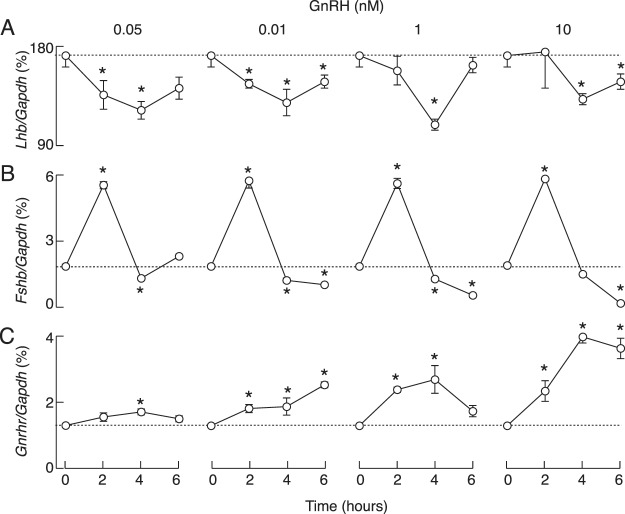
Concentration- and time-dependent effects of GnRH on *Lhb* (**A**) *Fshb* (**B**) and *Gnrhr* (**C**) expression in cultured pituitary cells. GnRH was applied in 0.05–10 nM concentrations. Asterisks indicate significant differences vs. zero time point control (dotted lines).

The lack of pronounced stimulation of *Lhb* expression during continuous 10 nM GnRH/D-Ala6 application, which is a pharmacological dose, prompted us to examine the full concentration dependence on GnRH of gene expression. To do this, static culture of pituitary cells were treated with 0.05, 0.1, 1, and 10 nM GnRH for 2, 4 and 6 h. The results of this concentration and time-course study, summarized in Fig. 3, confirmed the lack of a stimulatory effect and the presence of a minor inhibitory effect of GnRH on *Lhb* expression, with little effect of GnRH dose (Fig. 3A). In further parallelism with previous experiments, we observed a bidirectional effect of GnRH on *Fshb* expression, an early stimulatory and a sustained inhibitory effect (Fig. 3B). The time-course of GnRH-stimulated *Fshb* expression was comparable at all concentrations, with no effect of GnRH dose on the peak amplitude of responses, indicating maximal stimulatory action at picomolar GnRH concentrations. Conversely, downregulation of expression in the inhibitory phase was more pronounced with increasing GnRH concentration. The time course of GnRH-stimulated *Gnrhr* expression was also comparable at all GnRH concentrations, but the concentration dependence of this gene expression occurred later than the stimulatory component of the *Fshb* expression profile (Fig. 3C).

### *In vitro* expression of pituitary transcriptional factors controlling *Lhb* expression

**Figure 4:**
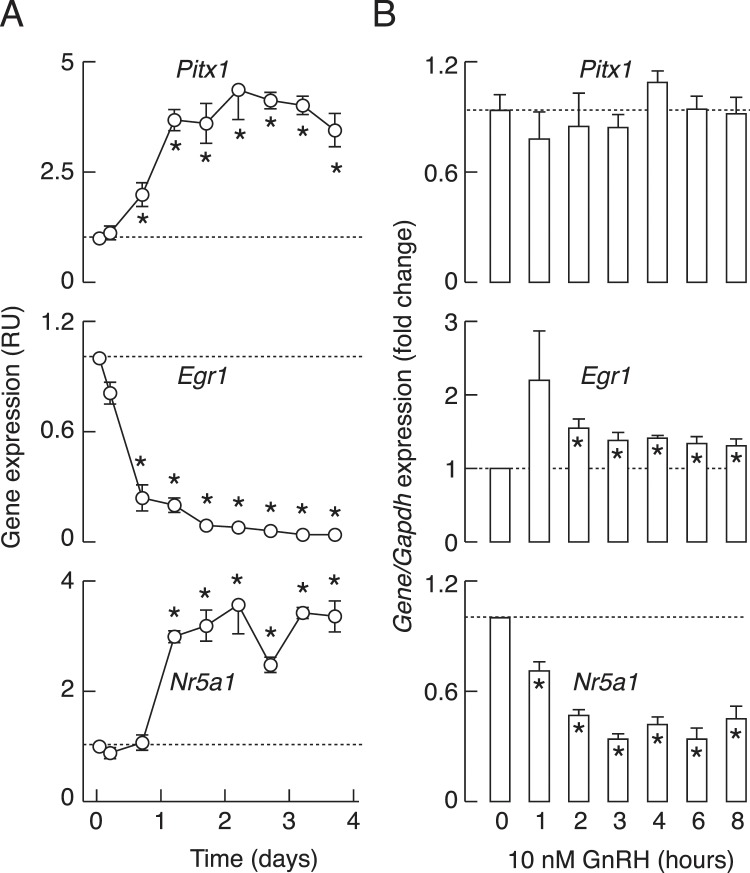
The expression patterns of transcriptional factor genes controlling *Lhb* in anterior pituitary cells cultured in the absence and presence of GnRH. Changes in *Pitx1*, *Egr1*, and *Nr5a1* expression in cells cultured for 96 h without GnRH (**A**) and in the presence of 10 nM GnRH, applied after 20 h culture in GnRH-free medium (**B**). Data points in A are mean ± SEM values from a representative experiment shown as relative units, and in B are normalized mean ± SEM values from four to nine experiments per time point, each done in quadruplicates. In all panels, asterisks indicate significant difference vs. zero time point controls.

To better understand the expression profile of *Lhb* expression, we examined the responses of *Pitx1*, *Egr1*, and *Nr5a1* to dispersion, culturing, and GnRH application. As stated in the Introduction, these transcription factors contribute to stimulation of *Lhb* expression. Dispersion/culturing of pituitary cells had dramatic effects on expression of these genes. There was a rapid, progressive, and deep decrease in *Egr1* expression, whereas *Pitx1* and *Nr5a1* expression was facilitated in the same experimental conditions (Fig. 4A). In contrast, continuous GnRH application had a rapid and profound inhibitory effect on *Nr5a1* expression, did not alter *Pitx1* expression, and induced a transient increase followed by a recovery to near baseline of *Egr1* expression, followed by a sustained plateau response (Fig. 4B). The opposite effects of culturing of cells without and with GnRH on *Egr1* and *Nr5a1* expression clearly indicates that this agonist plays an important role in their expression, stimulatory for *Egr1* and inhibitory for *Nr5a1*, whereas the expression of *Pitx1* was insensitive to GnRH and so appears to be controlled by other hypothalamic and/or intrapituitary factor(s).

**Figure 5:**
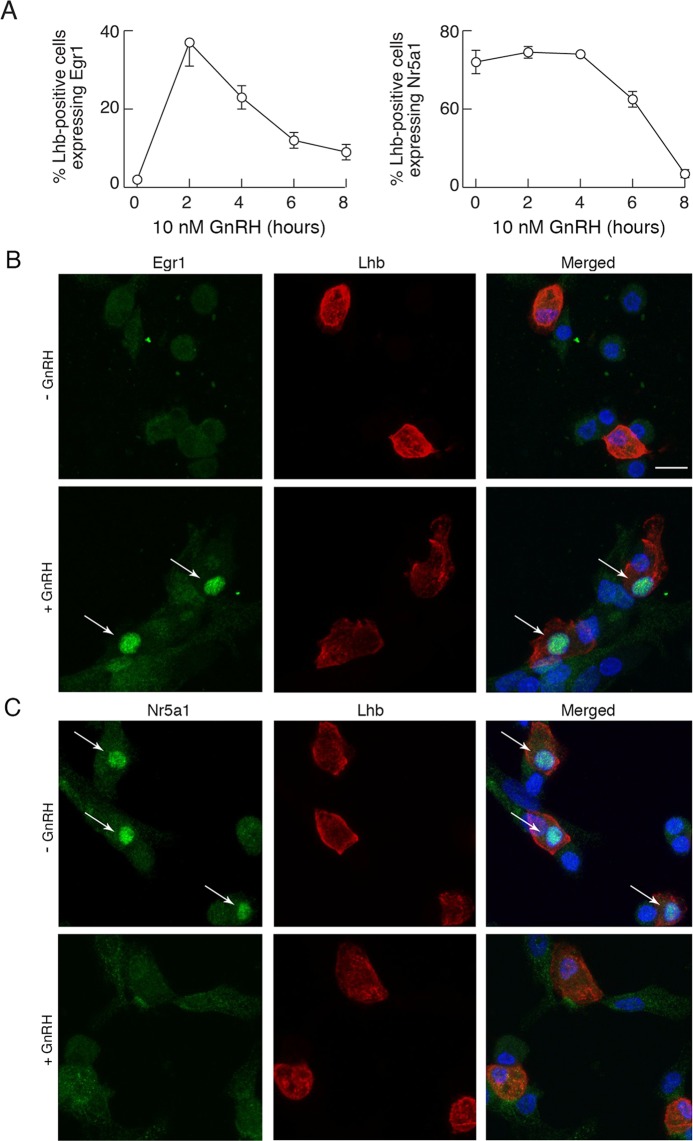
Immunofluorescence analysis of Egr1 and Nr5a1 expression in gonadotrophs. (**A**) Time course effect of GnRH treatment on Egr1 and Nr5a1 expression in Lhb-positive cells. (**B**,**C**) Representative images for immunocytostaining of Egr1- and Nr5a1-positive gonadotrophs. (**B**) Egr1 (green, left), Lhb (red, center), and their overlay (right) in untreated (top) and GnRH (10 nM/2 h) treated (bottom) pituitary cells. (**C**) Nr5a1 (green, left), Lhb (red, center), and their overlay (right) in untreated and GnRH (10 nM/8 h) treated (bottom) pituitary cells. Cell nuclei are stained with DAPI (blue). Arrows indicate Lhb-positive gonadotrophs that express either Egr1 or Nr5a1. Scale bars (applies to all images), 10 µm.

In further studies, the expression of Egr1 and Nr5a1 proteins was evaluated in anterior pituitary cells cultured overnight in horse serum-containing medium, washed in the morning and treated with BSA-containing medium with or without 10 nM GnRH. Following treatment, cells were double-stained with Egr1 or Nr5a1 and Lhb antibodies as described in *Methods: Immunocytochemistry*. Prior to GnRH treatment, Egr1 immunoreactivity was detected only in 2% of Lhb-positive cells. Following 2 h GnRH treatment, there was a rapid rise in expression of Egr1 in Lhb-positive cells (37%). The number of Egr1-positive cells decreased with further duration of treatment, but stayed above basal expression (23, 12 and 9% following 4, 6 and 8 h treatment, respectively; Fig. 5A, left). In contrast, about 65% of Lhb-positive cells also expressed Nr5a1 in untreated cultures and there was a significant decay in the fraction of Nr5a1 positive gonadotrophs after 6 h (45%) and 8 h (7%) of treatment with GnRH (Fig. 5A, right). Representative images for immune staining of Egr1- and Nr5a1-positive gonadotrophs are shown in Fig. 5B,C, respectively. The lack of effect of continuous GnRH application on stimulation of *Lhb* expression may be explained by the opposing responses in Egr1 and Nr5a1 expression after cell dispersion and during GnRH treatment. Also, the minor downregulatory effect of GnRH on basal *Lhb* expression indicates that the majority of basal *Lhb* expression is not GnRH controlled, in contrast to *Fshb* expression.

### *In vivo* effects of buserelin and cetrorelix acetate on gonadotroph gene expression

**Figure 6:**
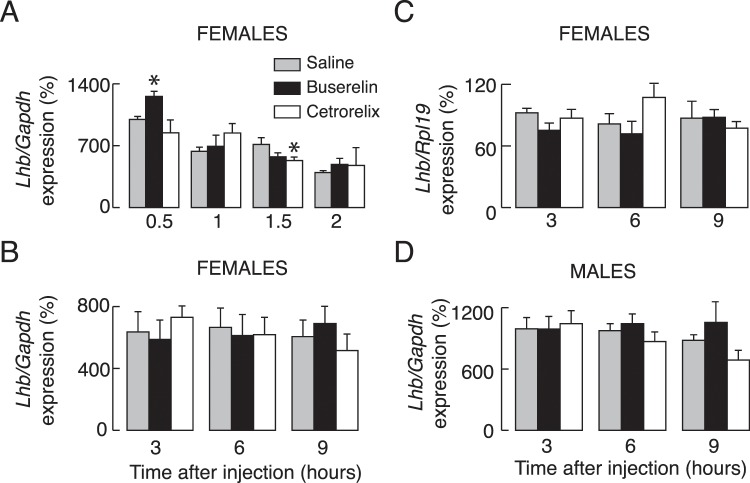
Effects of *in vivo* injection of a GnRH receptor agonist and antagonist on *Lhb* expression. Experiments were performed with prepubertal female and male rats. (**A**–**D**) Effects of injection of buserelin acetate, a GnRH receptor agonist (5 µg/0.4 ml per animal), cetrorelix acetate, a GnRH receptor antagonist (100 µg/0.4 ml per animals,) or solvent (0.4 ml PBS) intraperitoneally. Animals were sacrificed 0.5, 1, 1.5 and 2 h after injection (**A**) or 3, 6 and 9 h after injection (**B**–**D**). Values were normalized vs. *Gapdh* (**A**,**B**,**D**) or *Rpl19* (**C**).

**Figure 7:**
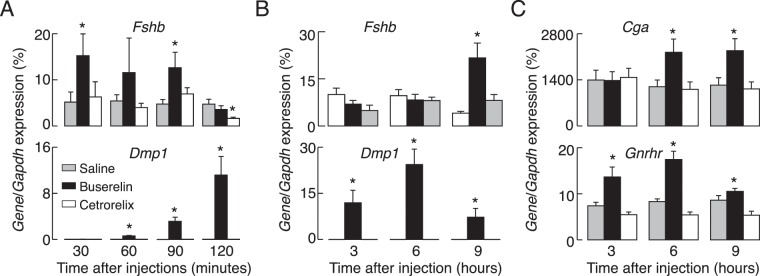
Effects of *in vivo* injection of a GnRH receptor agonist and antagonist on *Fshb*, *Cga*, *Gnrhr*, and *Dmp1* expression. Experiments were performed with prepubertal female rats sacrificed 0.5, 1, 1.5 and 2 h after injection (**A**) or 3, 6 and 9 h after injection (**B**,**C**).

Prepubertal female and male rats were injected with 5 µg of buserelin acetate, a GnRH receptor agonist, 100 µg of cetrorelix acetate, a GnRH receptor antagonist, or with vehicle (saline solution) intraperitoneally (Fig. 6). Animals were euthanized 0.5, 1, 1.5, and 2 h after injection (panel A), or 3, 6, and 9 h after injection (panels B-D), blood was collected for serum LH measurements and pituitary glands were removed for qRT-PCR analysis or LH tissue content measurements. We observed significant increases in *Lhb* expression only at one time point, 30 min after injection of buserelin acetate. Notice the lack of buserelin acetate effect on *Lhb* expression independent of the gene used for normalization of data, *Gapdh* (Fig. 6B) or *Rpl19* (Fig. 6C). However, buserelin acetate injection resulted in significant and transient increases in *Fshb, Gnrhr*, and *Dmp1* expression and a progressive increase in *Cga* expression. The timing for *Cga*, *Gnrhr*, and *Dmp1* expression *in vivo* was comparable to *in vitro* expression, whereas *Fshb* expression reached peak response already 30 min after the treatment, followed by decay in response and beginning of recovery of response after 9 h treatment (Fig. 7). Parallelism in the stimulatory actions of GnRH receptor agonists on gonadotropin subunit genes and *Gnrhr* expression *in vitro* and *in vivo* further supports the gene-specific expression pattern during continuous receptor activation.

### Independence of *Lhb* expression with respect to experimental conditions

It has been suggested that exposure of female pituitary cells to androgens is needed for establishing GnRH stimulus – transcriptional coupling^36^. To examine *in vitro* influence of androgens on *Lhb* expression, dispersed female pituitary cells were cultured overnight, washed and cultured for additional 24 h in the presence of 250 pg/ml dihydrotestosterone or 500 pg/ml testosterone, as previously described^37^. Cell were then washed and stimulated with 10 nM GnRH in the presence of androgens for 1–6 h. Figure S4 summarizes these investigations. Consistent with experiments without androgens (Fig. 2), GnRH also had a minor inhibitory effect on *Lhb* expression, but stimulated *Gnrhr* and *Fshb* expression during the first 6 h of exposure. The expression profiles for *Gnrhr* and *Fshb* were somewhat different in the presence of dihydrotestosterone and testosterone. Specifically, *Gnrhr* response was sigmoidal in the presence of dihydrotestosterone and linear in the presence of testosterone, and the duration of the upregulation phase of *Fshb* response was extended in the presence of testosterone (Fig. S4).

We also tested the validity of TaqMan probes and assay used for analysis. First, we used different TaqMan probes for *Lhb* and observed no difference in the expression pattern (data not shown). Second, we used static cultures of pituitary cells for 10 nM GnRH treatment (0 to 8 h) and SYBR-Green based assays with three different pairs of primers for evaluation of *Lhb* mRNA levels (Fig. S5A; Table S1). Consistent with the TaqMan assay, no stimulatory effect of GnRH on *Lhb* expression was observed by SYBR-Green assay. Finally, both TaqMan and SYBR-Green assays were used to evaluate *in vivo Lhb* expression after buserelin acetate and cetrorelix acetate injections; Fig. S5B summarizes results with TaqMan assay (*left*) and SYBR-Green assay (*middle*) and a significant correlation between values was obtained by the two assays (*right*), indicating the validity of both assays.

### Characterization of the LH secretory actions of GnRH receptor agonists *in vitro* and *in vivo*

**Figure 8:**
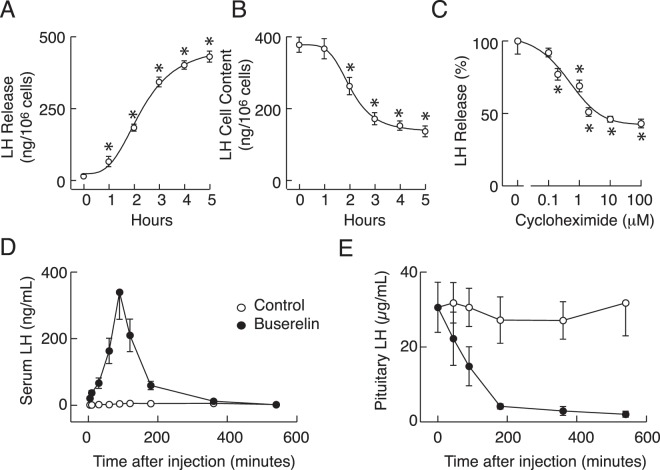
GnRH receptor agonist-stimulated LH secretion *in vitro* and *in vivo*. (**A**,**B**) Time-courses of GnRH-induced LH release (**A**) and depletion of intracellular LH content (**B**). After overnight incubation, cells were stimulated with 10 nM GnRH for 1–5 h. At the end of incubation periods, medium (**A**) and cell lysates (**B**) were collected for LH measurements. (**C**) Concentration-dependent effect of cycloheximide, a eukaryote protein synthesis inhibitor, on 10 nM GnRH-induced LH release following 4 h incubation (normalized values). Asterisks indicate significant differences vs. untreated cells. (**D**,**E**) *In vivo* effects of buserelin acetate on serum (**D**) and intrapituitary (**E**) LH. Data shown are mean ± SEM, N = 5 per time point. Panels D and E shows combined data from short-term and long-term treatment with buserelin acetate and control females (compare Figs. 6 and 7).

For secretory studies, 0.25 × 10^6^ cells per well were cultured in 24-well plates overnight, washed in the morning and stimulated with 10 nM GnRH for 1–5 h. At the end of incubation, medium was collected, and LH was measured in incubation medium and cellular extracts. Figure 8 illustrates the time-courses of GnRH-induced LH release (A) and depletion of intracellular LH content (B); the time-dependent increase in LH release was accompanied by a progressive decrease in hormonal cell content. We also examined effects of cycloheximide, an inhibitor of eukaryotic protein synthesis, on 10 nM GnRH-induced LH release during 4 h incubation. This compound caused a profound reduction of GnRH-stimulated LH secretion (C), indicating that high basal *Lhb* and *Cga* transcription leads to de novo LH synthesis.

*In vivo* injection of buserelin acetate also triggered LH secretion, as documented by measurements of serum LH concentration. There was a rapid increase in serum LH concentration, reaching a peak in secretion 90 min after injection, followed by a gradual decay (Fig. 8D). The spike phase temporally coincided with a rapid fall in pituitary LH tissue content (Fig. 8E). Nine hours after buserelin acetate treatment, pituitary LH tissue content was about 6% of that observed in controls. Taken together, these experiments indicate that during continuous treatment with high concentrations of GnRH receptor agonists there was no blockade of stimulus-secretion coupling and that basal *Lhb* expression and de novo LH synthesis are not sufficient to protect cells from depletion of the secretory pool.

## Discussion

Here we examined the dependence of gonadotropin subunit and GnRH receptor gene expression patterns on GnRH *in vitro* and *in vivo*. After cell dispersion and culturing in GnRH-free medium, we observed a rapid decay in *Fshb*, *Cga*, and *Gnrhr* expression, but only a small decay in *Lhb* expression. We also showed significant changes in expression of reference genes commonly used for quantification of relative expression by qRT-PCR in the same conditions, but not in response to GnRH treatment, necessitating careful analysis in the first 20 h of primary culture. In further agreement with the role of GnRH receptors in *Fshb*, *Cga*, and *Gnrhr* expression, *in vitro* continuous treatments with both GnRH and its degradation-resistant analog D-Ala6 stimulated the expression of these genes, but with gene-specific kinetics. *Fshb* expression was bidirectional, with a rapid but transient upregulation, followed by a sustained downregulation in expression below the basal value observed in untreated cell cultures. *Gnrhr* expression was also transient, but with a delay in peak expression and without downregulation of expression below basal levels. *Cga* expression was only upregulated with about 6–8 h-delay in beginning of rise in expression. In contrast, we did not consistently observe upregulation of *Lhb* expression, and we recorded only a minor and transient inhibition from basal *Lhb* expression levels during the continuous presence of GnRH receptor agonists. *In vivo* injected buserelin acetate also transiently stimulated *Fshb*, *Cga*, and *Gnrhr* expression without obviously affecting *Lhb* expression. The expression of *Dmp1*, another gonadotroph-specific gene, was comparable to *Gnrhr* expression kinetics.

To our best knowledge, we present the first study of the effects of culturing cells in GnRH-free medium on the time course of expression of these genes, starting immediately after cell dispersion. We also believe this is the first detailed time-course analysis of the effects of continuous GnRH receptor agonist treatments on gonadotropin subunit gene expression in the rat model. Others have observed up-regulation in *Fshb* expression 1 h after GnRH application in rat pituitary fragments^18^ and downregulation in expression of this gene in rat pituitary cells cultured for 8 and 24 h in the presence of GnRH^20^ and 24 h after continuous exposure to GnRH in cultured mouse pituitary cells^38^. In contrast to our results, Shupnik reported stimulation in *Lhb* expression in static culture of rat pituitary fragments, and rapid effects (within 30 min) on *Cga* expression^18^. However, Weiss *et al*. reported no stimulatory effect of continuous GnRH treatment of cultured rat pituitary cells on *Lhb* expression 4, 8, and 24 h after continuous stimulation with GnRH^20^. The lack of effect of GnRH treatment on *Lhb* expression was also observed in pituitary cells from ovariectomized rats^39^. In contrast to gonadotropin subunit genes, previous experiments have well clarified that expression of *Gnrhr* decays during prolonged culturing in the absence and presence of GnRH^26,40,41^. GnRH treatment also elevates *Dmp1* expression *in vitro* with similar kinetics and concentration dependence^27,42^. The same conclusion was reached in this study, which gave us confidence about the reproducibility of *in vitro* experiments and the validity of the novel conclusions for expression of gonadotropin subunit gene expression during continuous *in vitro* application.

Our experiments lead to at least four major conclusions: 1. The lack of ability of continuous GnRH treatment to modulate *Lhb* expression is consistent with the hypothesis that a pulsatile pattern of GnRH application may be a pre-requisite for detecting the upregulation in *Lhb* expression, which has been previously reported^16–18^. 2. Continuous GnRH treatment not only transiently stimulates *Fshb* expression, but also downregulates basal *Fshb* expression. FSH is known to be predominantly secreted by constitutive exocytosis. Consequently, the intrapituitary *Fshb* and circulating FSH levels are often highly correlated; i.e. *Fshb* expression is a good indicator of the secretory profile^21^. Thus, it is reasonable to conclude that blockade of *Fshb* expression by continuous GnRH treatment accounts for downregulation of FSH release in such treatments. 3. The difference in the peak response times for *Fshb*, *Gnrhr*/*Dmp1*, and *Cga* could provide a rationale for the observed differences in the GnRH pulse frequency in regulation of expression of these *Fshb* (low), *Gnrhr* (high), and *Cga* (high or continuous). This motivates further experiments to clarify the differences in the intracellular messengers and/or transcriptional factors controlling their expression. 4. Such divergent patterns of expression of gonadotropin subunit and GnRH receptor genes strongly argue against desensitization of GnRH receptors during continuous treatment. This is consistent with previous findings that the type I mammalian GnRH receptor does not desensitize, in contrast to other G protein-coupled receptors^43^ and non-mammalian forms of this receptor^44^. Our results also indicate that receptor signaling pathways are functional during continuous stimulation, accounting for a prolonged upregulation and downregulation of *Cga* and *Fshb* expression, respectively, and continuous LH secretion.

To understand why no stimulatory action of GnRH on *Lhb* expression was observed during continuous receptor activation, we studied the expression pattern of three transcription factors, Egr1, Nr5a1, and Pitx1, which have been suggested to be critical for controlling basal and/or GnRH receptor-regulated *Lhb* expression^45^. *Egr1* null female mice are infertile and lack expression of *Lhb*^24^. This gene is induced rapidly by GnRH in immortalized gonadotrophs^46^ and cultured rat pituitary cells^27,47^. The action of Egr1 protein on *Lhb* transcription depends on Nr5a1 and Pitx1^25,48^, and such a pattern of regulation is largely conserved among mammals^49^. Consistent with this model, it has been suggested that GnRH stimulates *Nr5a1* expression^50^. However, others reported inhibitory effects of GnRH on expression of this gene^51^. In our preparation, *Egr1* and *Pitx1* were expressed in all subpopulations of pituitary cells, including gonadotrophs, whereas Nr5a1 was detected only in gonadotrophs^35^. Here we showed that *Egr1* expression rapidly declined and that protein expression was observed only in 2% of cells after 20 h incubation in GnRH-free medium. The loss of Egr1 proteins in cultured gonadotrophs was not accompanied by dramatic changes in basal *Lhb* expression. The subsequent addition of GnRH only partially restored the expression of this gene and protein. Furthermore, *Nr5a1* gene and protein expression was downregulated during continuous GnRH application. Thus, it is reasonable to speculate that basal *Lhb* expression is independent of the presence of Egr1 and that pulsatile GnRH application is required for the proper expression of Egr1/Nr5a1 and their coupling to *Lhb* expression.

The presence of high basal *Lhb* expression combined with the lack of sustained downregulation in *Lhb* expression during continuous GnRH application raised a question concerning the mechanism accounting for downregulation of LH secretion. It is well established that LH is predominantly secreted by regulated exocytosis^22^, and that GnRH is a very potent secretagogue^1^, which led us to examine the status of LH secretion under conditions used for studies on *Lhb* expression. We observed downregulation of LH secretion *in vitro* during sustained application of high concentrations of GnRH despite sustained *Lhb* expression. We also observed almost complete depletion of the LH secretory pool in gonadotrophs *in vivo* after injection of buserelin acetate despite elevated *Lhb* expression. These results indicate that downregulation of secretion occurs downstream of *Lhb* expression, probably at the level of de novo LH synthesis and/or maintenance of the secretory pool. It has already been suggested that translational control of gene expression could provide a rationale for some aspects of GnRH receptor signaling and secretion that could not be explained by changes at the level of transcription^52^. In that respect, it is illustrative that cycloheximide, an inhibitor of translational elongation, has a profound and time-dependent effects on LH release. These findings raise the possibility that pulsatile GnRH provides a mechanism for sustained LH release by allowing sufficient time for de novo LH synthesis to replenish the LH secretory pool. Further experiments are needed to clarify the status of LH secretory pool during sustained application of variable GnRH concentrations, and the kinetics of repletion of the secretory pool after removal of GnRH receptor agonists.

## Supplementary information

Supplementary Information
