# Supplementary material for: Divergent expression patterns of pituitary gonadotropin subunit and GnRH receptor genes to continuous GnRH in vitro and in vivo
Source: Sci Rep. 2019 Dec 27;9:20098. doi: 10.1038/s41598-019-56480-1 (PMC6934515; doi:10.1038/s41598-019-56480-1)
Supplement: Supplementary file 1 — Supplementary Information [file 41598_2019_56480_MOESM1_ESM.docx]

Supplementary Material

**Divergent expression patterns of pituitary gonadotropin subunit and GnRH receptor genes to continuous GnRH in vitro and in vivo**

**Marija M. Janjic^1,2,^*, Rafael M. Prévide^1,^*, Patrick A. Fletcher^3^, Arthur Sherman^3^, Kosara Smiljanic^1^, Daniel Abebe^1^, Ivana Bjelobaba^1,2^, and Stanko S. Stojilkovic^1, a^**

From the ^1^*Eunice Kennedy Shriver* National Institute of Child Health and Human Development and ^3^Laboratory of Biological Modeling, National Institute of Diabetes, Digestive and Kidney Diseases, National Institutes of Health, Bethesda, MD 20892, USA, and ^2^Institute for Biological Research *Sinisa Stankovic* - National Institute of Republic of Serbia, University of Belgrade University of Belgrade, 11000 Belgrade, Serbia.

**
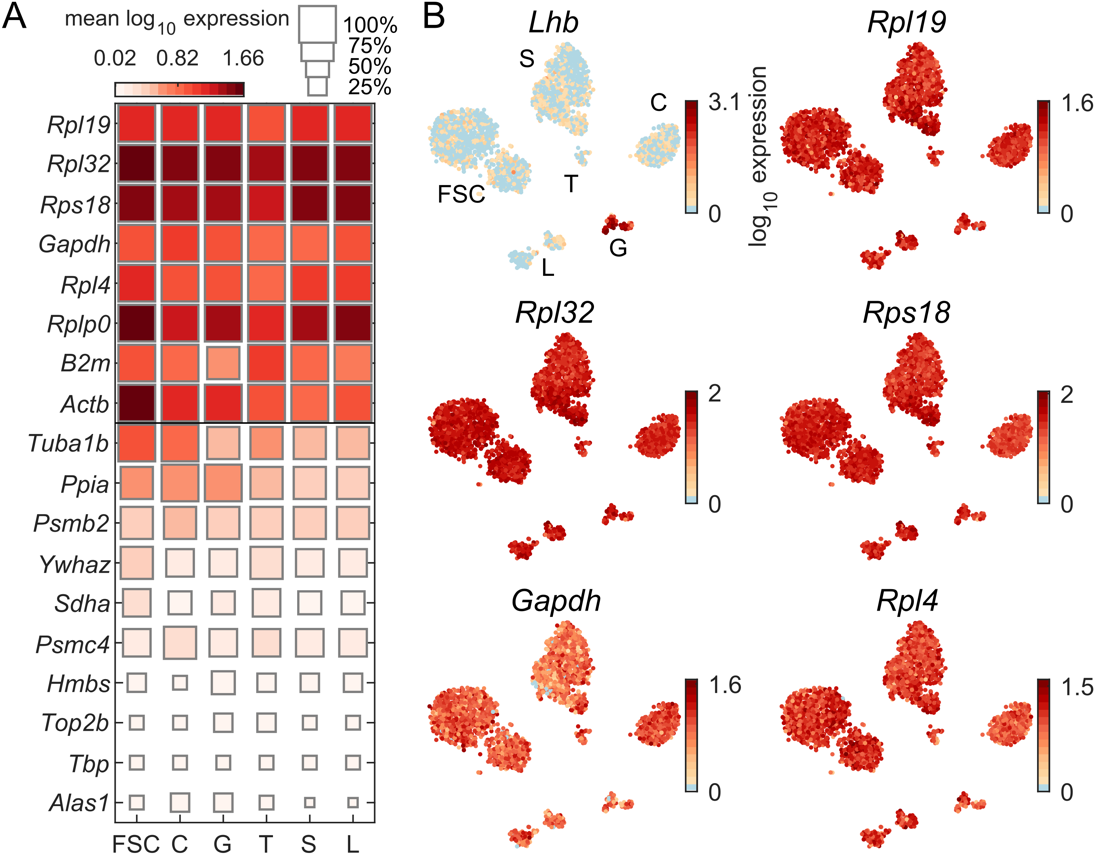
**

**Figure S1.** **Single cell expression patterns of candidate reference genes.** (A) Dot plot showing mean log_10_ expression (marker color) and percentage of cells expressing (marker size) reference genes. Genes expressed in >90% of cells are shown above the black horizontal line, sorted by uniformity of expression across cell types. (B) tSNE plots showing the pattern of expression of *Lhb* and the top five candidate reference genes among pituitary cells: FSC, folliculstellate cells; C, corticotrophs; G, gonadotrophs; T, thyrotrophs; S, somatotrophs; L, lactotrophs. To assess uniformity of expression among pituitary cell types, we computed the sum of relative error between the mean expression per cell type and the mean expression among all pituitary cells. Genes with lower total relative error were considered to have higher uniformity of expression. These and data shown in Fig. S2 were derived from data published in the NCBI Gene Expression Omnibus, GEO Series accession number GSE132224. The data processed and analyzed as described in Fletcher PA, Smiljanic K, Previde RM, Iben J, Li T, Rokic MB, Sherman A, Coon SL and Stojilkovic SS (2019). Cell Type- and Sex-Dependent Transcriptome Profiles of Rat Anterior Pituitary Cells. Front Endocrinol (Lausanne) 10:623. doi: 10.3389/fendo.2019.00623.


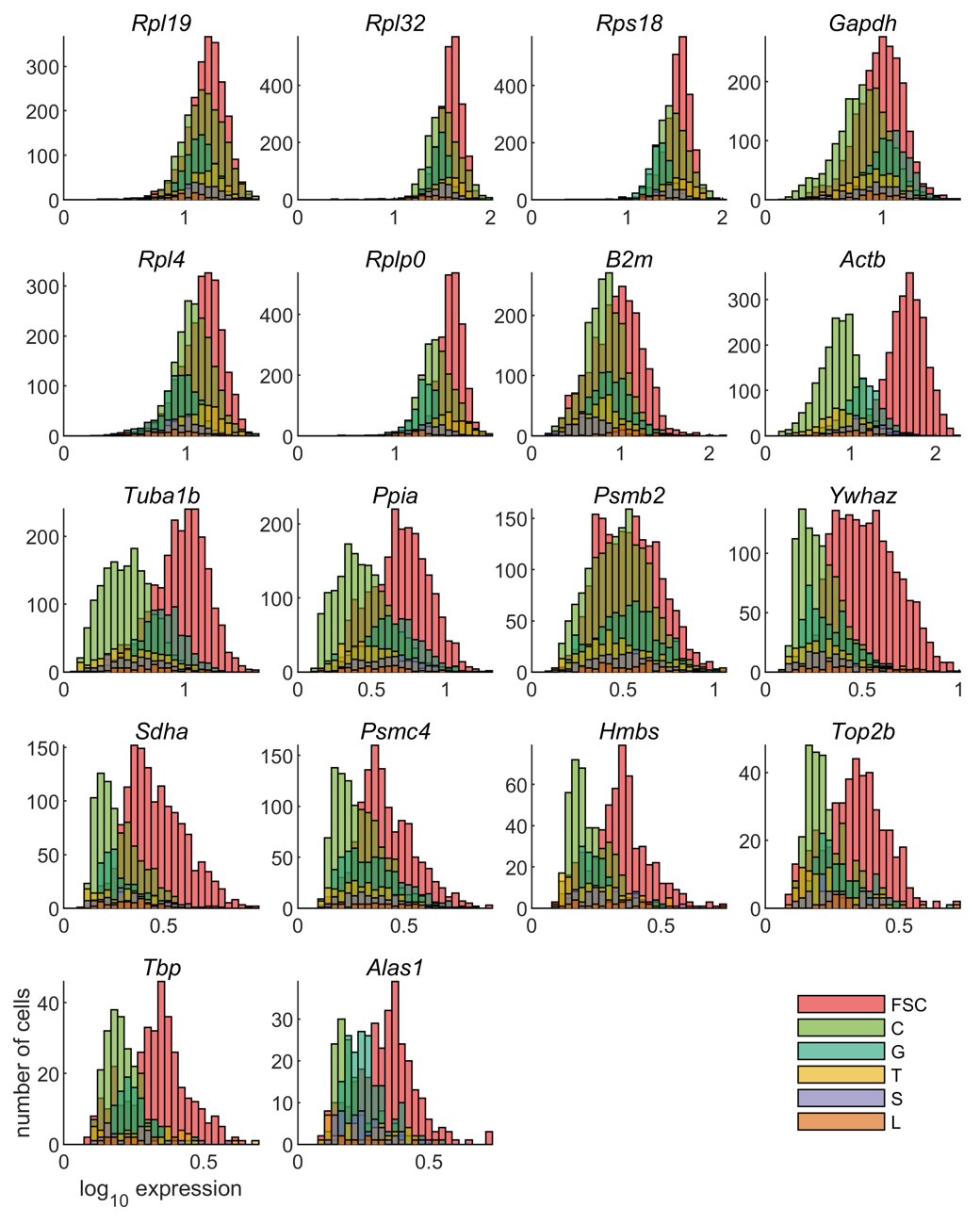


**Figure S2.** **Distributions of candidate reference gene expression in anterior pituitary cell types, measured by single cell RNA sequencing in freshly dispersed cells.** Cells with zero counts are not shown. Cell types: FSC, folliculostellate cells; C, corticotrophs; G, gonadotrophs; T, thyrotrophs; S, somatotrophs; L, lactotrophs.

**Figure S3. Effects of anterior pituitary cell dispersion and culturing on expression of candidate reference genes.** (A) Increase in expression of *Gapdh, Rpl19*, and *Rps18* during the first 24 h on incubation after cell dispersion. Cells were seeded in 24-well plates and cultured in M199 supplemented with bovine serum albumin (BSA), fetal bovine serum (FBS), or horse serum (HS). (B) Transient nature of increase in expression of candidate reference genes. Cells were cultured in BSA-containing M199. (C and D) The lack of effect of 10 nM GnRH on reference gene expression during 8 h (C), 2 h (D, top) and 60 h (D, bottom) incubation. After dispersion, pituitary cells from females were cultured in M199 with HS. After 20 h incubation, medium was replaced with 0.1% BSA and 10 nM GnRH-containing M199.

**Figure S4**. **Characterization of GnRH-induced marker genes expression in female pituitary cells bathed in androgen-containing medium.** After 20 h incubation, 250 pg/ml dihydrotestosterone (DHT; left panels) or 500 pg/ml testosterone (right panels) were added and cells where cultured for an additional 24 h, washed, and stimulated with 10 nM GnRH for 0 - 6 h in the presence of androgens. Charcoal-stripped serum was not used in this or other experiments.

**Figure S5. Comparison of TaqMan and SYBR-Green assays in evaluation of *Lhb* expression in cultured female pituitary cells.** (A) SYBR-Green analysis of *Lhb* expression in static cultures. Three sets of primers (I – III; Table S1) were used for analysis of *Lhb* transcripts in cultured cells. TaqMan analysis of the same samples also revealed the inability of GnRH to induce *Lhb* (data not shown). (B) TaqMan (left) and SYBR-Green (middle) analyses of in vivo expression of pituitary *Lhb*, 3, 6 and 9 h after injection of buserelin acetate (black bars), cetrorelix acetate (white bars) and solvent (gray bars). Right panel illustrates correlations in values obtained by two assays. R, Pearson’s coefficient of correlation.

**Table S1.**Primers sequences (Integrated DNA Technologies, Skokie, IL) used for SYBR-Green quantitative RT-PCR analysis.

| **Gene** | **Gene Accession Number** | **Primer Sequence** | **Transcript length** |
| --- | --- | --- | --- |
| *Gapdh* | NM_053294.4 | F:5’ – CAACTCCCTCAAGATTGTCAGCAA – 3’  R: 5’ – GGCATGGACTGTGGTCATGA – 3’ | 118 |
| *Lhb* (1) | NM_012858.2 | F:5’ – AGCATGGTTCGAGTACTGCC – 3’  R: 5’ – GACCCCCACAGTCAGAGCTA– 3’ | 190 |
| *Lhb2* (2) | NM_001033975.1  NM_012858.2 | F:5’ – AGCATGGTTCGAGTACTGCC – 3’  R: 5’ – GACCCCCACAGTCAGAGCTA– 3’ | 190 |
| *Lhb* (3) | NM_001033975.1  NM_012858.2 | F:5’ – CCTGGCTGCAGAGAATGAGT – 3’  R: 5’ – GTAGGTGCACACTGGCTGAG– 3’ | 132 |
